# The bacterial community associated with the sheep gastrointestinal nematode parasite *Haemonchus contortus*

**DOI:** 10.1371/journal.pone.0192164

**Published:** 2018-02-08

**Authors:** Gajenathirin Sinnathamby, Gemma Henderson, Saleh Umair, Peter Janssen, Ross Bland, Heather Simpson

**Affiliations:** 1 Institute of Veterinary, Animal and Biomedical Sciences, Massey University, Palmerston North, New Zealand; 2 AgResearch Ltd, Palmerston North, New Zealand; University of British Columbia, CANADA

## Abstract

Culture-independent methods were used to study the microbiota of adult worms, third-stage larvae and eggs, both in faeces and laid *in vitro*, of *Haemonchus contortus*, a nematode parasite of the abomasa of ruminants which is a major cause of production losses and ill-health. Bacteria were identified in eggs, the female reproductive tract and the gut of adult and third-stage larvae (L3). PCR amplification of 16S rRNA sequences, denaturing gradient gel electrophoresis (DGGE) and clone libraries were used to compare the composition of the microbial communities of the different life-cycle stages of the parasites, as well as parasites and their natural environments. The microbiomes of adult worms and L3 were different from those in the abomasum or faeces respectively. The *H*. *contortus* microbiota was mainly comprised of members of the phyla Proteobacteria, Firmicutes and Bacteroidetes. Bacteria were localised in the gut, inside eggs and within the uterus of adult female worms using the universal FISH Eub338 probe, which targets most bacteria, and were also seen in these tissues by light and transmission electron microscopy. *Streptococcus/Lactococcus* sp. were identified within the distal uterus with the probe Strc493. Sequences from the genera *Weissella* and *Leuconostoc* were found in all life-cycle stages, except eggs collected from faeces, in which most sequences belonged to *Clostridium* sp. Bacteria affiliated with *Weissella*/*Leuconostoc* were identified in both PCR-DGGE short sequences and clone libraries of nearly full length 16S rRNA bacterial sequences in all life-cycle stages and subsequently visualised in eggs by fluorescent *in situ* hybridisation (FISH) with group-specific probes. This strongly suggests they are vertically transmitted endosymbionts. As this study was carried out on a parasite strain which has been maintained in the laboratory, other field isolates will need to be examined to establish whether these bacteria are more widely dispersed and have potential as targets to control *H*. *contortus* infections.

## Introduction

Bacteria have developed symbiotic relationships with multicellular host organisms; these range from fatal pathogenic infections and parasitism to commensalism (to the advantage of the symbiont) or mutualism (which benefits both partners) [1–3]. The boundaries between these associations are not always distinct and may change with the physiology of the bacteria or host. Symbionts may be located externally or internally (ectosymbionts on the host surface or endosymbionts living within tissues), be obligate primary endosymbionts or facultative secondary symbionts and be transmitted vertically or acquired anew by each generation of host. The most extensively researched symbionts of nematodes are those in filarial and plant-parasitic nematodes and the entomopathogenic nematodes (EPNs), because of their medical and agricultural significance, although there are many other unusual bacterial-nematode symbioses of biological interest.

Most symbionts of nematodes are likely to be associated with either the gut or external surfaces and to be commensals or mutualists contributing principally to host metabolism. There is an interesting nutritional symbiosis between bacteria and the gutless stilbonematid marine worms, which obtain nutrition from external lawns of densely packed ectosymbiotic bacteria, in many cases a species-specific monoculture [4–7]. The focus of many studies of the microbiome of *Caenorhabditis elegans* has been differentiating those species which constitute a food source or are mutualists from species which are potential pathogens of either the nematode or other organisms for which the nematode acts as a vector of the bacteria [8–12]. The microbiomes of the free-living nematodes *C*. *elegans*, *Caenorhabditis remanei* [13], *Acrobeloides maximus* [14] and five grassland soil species [15] were different from those in their environments, less diverse and dominated by Proteobacteria. As in other multicellular hosts, the microbiome was not the same in all individuals and dependent on diet, genetics [13] and the presence of pathogens [12].

Nematodes symbionts can be exploited to control some important agricultural insect pests. The EPNs of the genera *Steinernematidae* and *Heterorhabditidae* use their γ-proteobacterial *Xenorhabdus* sp. symbionts, which colonise the intestine of infective third-stage larvae (L3) to kill host insects to continue the nematode reproductive cycle [16]. After entry of the L3 into the haemolymph of an insect, the bacteria are released and induce a fatal septicaemia. The EPNs reproduce for 2–3 generations within the insect, after which L3 take in symbionts and emerge into the soil to infect another insect and continue the life-cycle [2,17–19]. In contrast, the nematodes themselves may be the pests which may be able to be controlled via their symbionts. This has been proposed for the Verromicrobial endosymbionts of plant-parasitic nematodes, which are destructive cyst-forming pests of soybeans, potatoes and peas [20]. These *Candidatus* endosymbionts are specific for each species of nematode and are vertically transmitted by the females [21,22].

Many, but not all, filarial nematode parasites of humans and animals carry the maternally-transmitted essential endosymbiont *Wolbachia pipientis* [23–25], which is susceptible to antibiotic therapy. In these nematodes, the bacteria are necessary for normal worm embryogenesis, development and adult survival [26–30] and contribute to metabolism [31]. *Wolbachia* are transmitted vertically in eggs, thence to the gut of L3, developing lateral cords and adult female ovary, but not the male testis [32]. Other clades of *W*. *pipientis* are essential in many insects, but there are no confirmed reports of *Wolbachia* in other nematodes, apart from the plant-parasitic nematode *Radopholus similis* [33].

Gastrointestinal nematodes of farmed livestock cause health and welfare issues and enormous economic losses in pasture-based grazing systems [34–36]. *H*. *contortus*, a blood-sucking gastric parasite of ruminants, may cause a life-threatening disease in a severe infection [37,38]. Anthelmintics are currently the method of choice to control gastrointestinal parasites [39], however, the rapid spread of drench resistance [40] is driving the search for alternatives to chemical treatment, such as biological control via essential bacterial symbionts. *H*. *contortus* adult female worms lay eggs, which pass out in the faeces and hatch into first-stage larvae (L1) under favourable warm and moist conditions. L1 develop and moult on the pasture into L2, both stages feeding on the faecal bacteria, then L2 moult into L3, the infective and non-feeding stage, which retains the L2 cuticle as a protective sheath. After L3 are consumed by an appropriate host, they exsheathe (shed the L2 cuticle) in the rumen, move down to the abomasum and enter the lumen of gastric glands, where they develop and after 2–4 days emerge either as L4 or immature adult worms. The very fecund female *H*. *contortus* lay 5,000–10,000 eggs per day, beginning after 12–15 days, although this is variable [38,41].

The aim of the present study was to investigate the bacteria associated with *H*. *contortus* using DNA fingerprinting. First, the communities in cultured L3, adult worms and eggs (either recovered from eggs or from adult worms) were compared with those in their environments by PCR amplification of 16S rRNA sequences and separation of products by denaturing gel electrophoresis (DGGE). Detailed phylogenetic evolutionary relationships were then determined to identify the bacteria present in the different life-cycle stages. Bacteria were located within the parasites by light microscopy (LM), transmission electron microscopy (TEM) and fluorescence in situ hybridisation (FISH).

## Results/Discussion

The bacteria associated with *H*. *contortus* were identified using PCR amplification of 16S rRNA sequences from eggs, L3 and adult worms.

### Bacterial communities are not identical in parasites and their environment

PCR-DGGE analysis of 16S rRNA sequences showed that the bacterial communities in male and female adult worms generated very similar DGGE band patterns (S1 Fig). Poor separation of sequences from abomasal contents did not allow comparison of the communities in adult worms and contents, however, abomasal bacteria appeared to adhere to the cuticle, as band patterns from worms set in agar blocks and allowed to migrate out and subsequently cleaned by washing with 4% sodium hypochlorite differed from bands from manually collected worms (S2 Fig). Even identical band patterns for two samples are indicative only of similar bacterial communities, as 16S rRNA sequences which are different in G+C content and/or belong to different bacterial species can be isolated in one band and, conversely, sequences belonging to one species can be present in more than one band, due to the presence of multiple rrn operons.

L3 and eggs were subsequently also cleaned with sodium hypochlorite in an attempt to remove adherent bacteria. The effects of the recovery and cleaning processes on the number of DGGE bands associated with L3 are shown in S3 Fig. The L3 communities did not simply reflect those in the faeces in which they were cultivated and the band pattern was altered by a combination of exsheathing and washing (S3 Fig). Some bacteria present in the adult worm gut may have persisted throughout the parasite life-cycle from development of L1 to L2, which feed on faecal bacteria, on to non-feeding L3 and parasitic adult worms.

**Fig 1:**
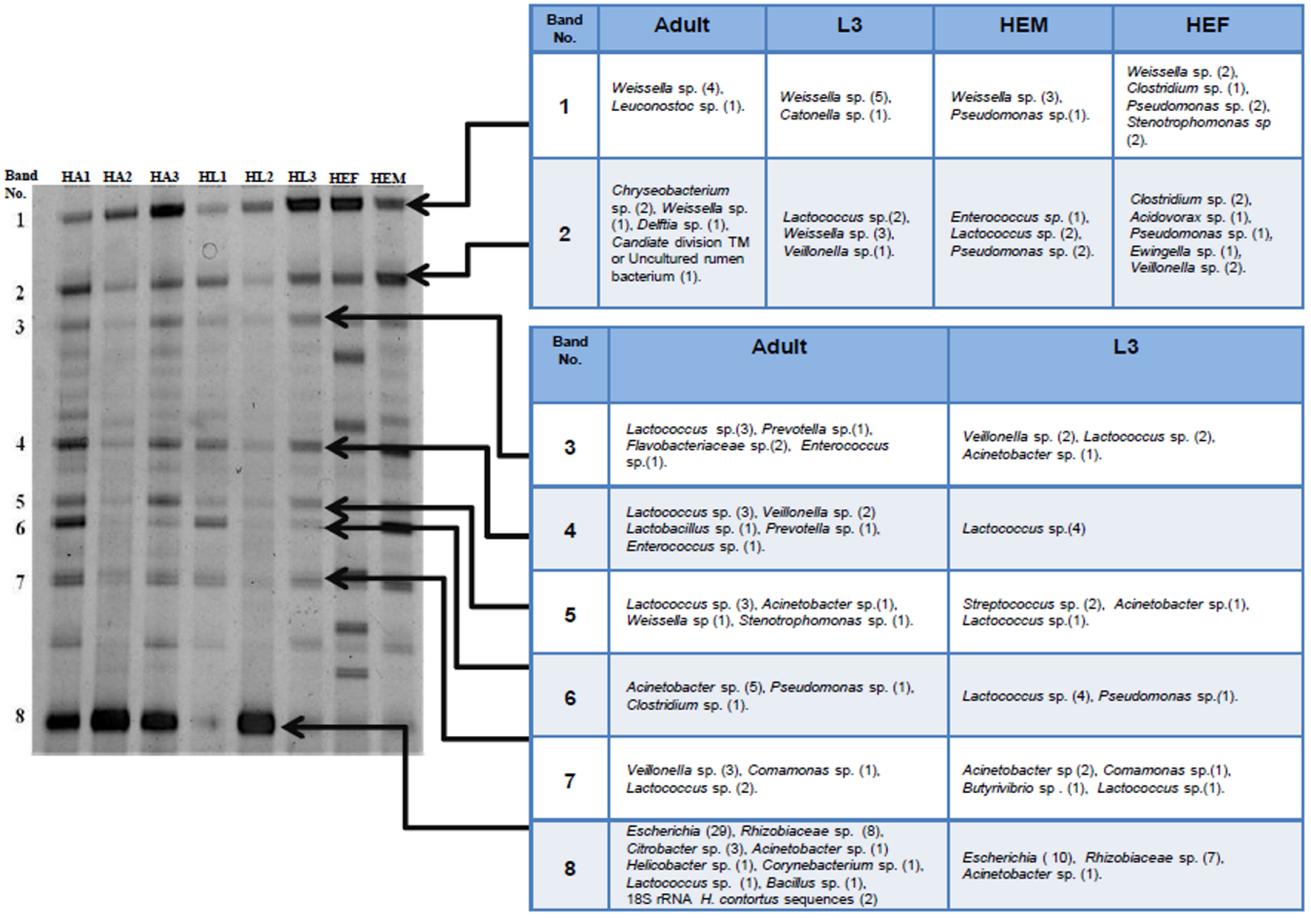
A representative DGGE gel of PCR amplified products generated from DNA of *H*. *contortus* adult worms (HA), L3 (HL) and eggs (HEM: *in vitro* laid eggs and HEF: eggs collected from faeces) used for sequencing the bands of interest (left). The phylogenetic affiliations of sequences obtained from the individual DGGE bands are shown in the table (right).

Eggs would not be expected to have many bacteria associated with them if the eggshells had been effectively cleaned. This was clearly not the case after sodium hypochlorite washing, probably because bacteria or DNA were firmly attached to the carbohydrate coat present on the surface of the eggs [42]. Sheep faecal bacteria appeared to contaminate eggs collected from the faeces, as there were intense bands in DGGE gels of sequences from eggs extracted from faeces that were not present in eggs laid *in vitro* (Fig 1). These bands were subsequently found to contain sequences of *Clostridium* sp., which are typical bacteria associated with faeces [43–46]. The band patterns from *in vitro* laid eggs and the adult females that laid them were similar (Fig 1, lanes HA1 and HE2), suggesting these eggs acquired bacteria from gut contents of the mother during egg-laying.

### Preliminary identification of bacteria in adult worms, L3 and eggs

Adult worms, L3 and eggs generally produced similar DGGE bands consisting of 7 major bands common to all three stages and an eighth not present in eggs (Fig 1). These bands were excised and ~190bp sequences amplified with the primer set 338f and 518r, which is widely used for analysing bacterial sequences by the DGGE fingerprinting method [47,48]. This allowed preliminary identification of bacterial 16S RNA, although the ~190bp sequences generated were too short for detailed phylogenetic analysis and identification of species. A total of 44 and 21 bacterial sequences were obtained from these 7 bands in 3 separate gels of adult worms and L3 respectively and 23 sequences from bands 1 and 2 from eggs. An eighth band, not present in eggs, yielded 45 bacterial sequences from adult worms and 18 from L3; these were mainly *Escherichia* (39) and *Rhizobia* sp. (15). The phylogenetic affiliations of the sequences are shown in Fig 1 and summarised in Table 1.

**Table 1:** Summary of phylogenetic affiliations of ~190bp bacterial sequences obtained from DGGE bands from *H*. *contortus* adult worms, L3 and eggs, both extracted from faeces and laid *in vitro*.

Bacteria					Adult	L3	Eggs
Phylum	Class	Order	Family	Genus			
**Firmicutes**	Bacilli	Lactobacillales	Enterococcaceae	*Enterococcus*	2		1
			Streptococcaceae	*Lactococcus*	11	14	2
				*Streptococcus*		2	
			Leuconostocaceae	*Leuconostoc*	1		
				*Weissella*	6	8	5
			Lactobacillaceae	*Lactobacillus*	1		
**Total lactic acid bacteria (LAB)**					**21**	**24**	**8**
	Clostridia	Clostridiales	Clostridiaceae	*Clostridium*	1		3
			Lachnospiraceae;	*Catonella*		1	
				*Butyrivibrio*		1	
			Veillonellaceae	*Veillonella*	5	3	2
**Total Firmicutes**					**27**	**29**	**13**
**Proteobacteria**	Gammaproteobacteria	Pseudomonadales	Moraxellaceae	*Acinetobacter*	6	4	
			Pseudomonadaceae	*Pseudomonas*	1	1	6
		Xanthomonadales	Xanthomonadaceae	*Stenotrophomonas*	1		2
		Enterobacteriales	Enterobacteriaceae	*Ewingella*			1
	Betaproteobacteria	Burkholderiales	Comamonadaceae	*Delftia*	1		
				*Acidovorax*			1
				*Comamonas*	1	1	
**Total Proteobacteria**					**10**	**6**	**10**
**Bacteroidetes**	Bacteroidetes	Bacteroidales	Prevotellaceae	*Prevotella*	2		
				Un-cultured rumen bacterium	1		
	Flavobacteriales	Flavobacteriaceae	Flavobacteriaceae	*Flavobacteriaceae* or *Chryseobacterium*	4		
**Total Bacteroidetes**					**7**		
**Total sequences**					**44**	**35**	**23**

About half of the ~190bp sequences were matched to the phylum Firmicutes and the rest were consistent with sheep gut or ubiquitous environmental bacteria. Lactic acid bacterial and Proteobacterial sequences dominated those identified from adult worms, L3 and eggs. Alpha-, beta- and gamma-proteobacteria have been identified in the microbiomes of other nematodes [13,15,49,50] and are also ubiquitously present in aquatic environments [51] and therefore may be either commensals or contaminants acquired from the parasite environment. These bacteria are unlikely to have originated from laboratory reagents, which were screened for contaminants.

### Phylogenetic analysis of bacterial sequences from adult worms, L3 and eggs

More detailed phylogenetic evolutionary relationships were determined for longer bacterial 16S rRNA sequences amplified from DNA extracted from adult worms, L3 and eggs. Clone libraries were constructed using the universal bacterial primer set (27f and 1492r) to amplify nearly complete length (~1400bp) 16S rRNA sequences and for ~1000bp sequences amplified by 27f and 1040firmR Firmicutes-specific primers (Table 2).

**Table 2:** Primer pairs or FISH probes used for PCR-DGGE, DNA fingerprinting and location using FISH of bacteria associated with *H contortus*.

Protocol	Primer/FISH probe	PCR amplified fragment size	Sequences (5'-3')	Reference
DGGE	338f*	~180bp	ACWCCTACGGGWGGCWGC	Lane et al. (1991)
	518r		ATTACCGCGGCTGCTGG	Muyzer et al. (1993)
Clone library: Universal primers	27f	~1400bp	GAGTTTGATCMTGGCTCAG	Modified from Lane et al. (1991)
	1492r		GGYTACCTTGTTACGACTT	
Clone library: Firmacutes specific	27f	~1000bp	GAGTTTGATCMTGGCTCAG	From Lane et al. (1991)
	1040firmR		ACCATGCACCACCTGTC	Meier et al. (1999)
FISH: most bacteria	EUB338 (Cy3 or FITC)	16S, 338–355	GCTGCCTCCCGTAGGGT	Amman et al. (1990)
FISH: negative control	Non-EUBb338 (Cy3 or FITC)	16S, 338–355	ACTCCTACGGGAGGCAGC	Wallner et al. (1993)
FISH: Lactic acid bacteria	Lab158 (Cy3 or FITC)	16S, 176–195	GGTATTAGCAYCTGTTTA	Harmsen et al. (2002)
FISH: *Weissella* sp,	Wgp (Cy3)	16S, 150–171	TTATCCCCYRCTAAGAGGTAGG	Collins et al. (1993)
FISH: *Weissella* sp,	S-G-Wei-0121-a-S-20 (Cy3)	16S, 141–160	TAAGAGGTAGGTTTCCCG	Jang et al. (2002)
FISH: Most *Streptococcus* sp., some *Lactococcus* sp	Strc493 (Cy3 or FITC)	16S, 493–511	GTTAGCCGTCCCTTTCTG	Franks et al. (1998)
FISH: Some Alphaproteobacteria	ALF73a (Cy3)	23S, 2043–2059	TTCCGTCTAACCGCGGG	Manz et al. (1992)
FISH: Betaproteobacteria	Beta1(Cy3)	16S, 359–378	CCCATTGTCCAAAATTCC CC	Ashelford et al. (2002)
FISH: *Stenotrophomonas maltophilia*	SteMal439 (Cy3)	16S, 439–458	GCT GGA TTT CTT TCC CAA CA	Piccini et al. (2006)

*GC clamp added to the 5' end of the primer, 5'CGC CCG CCG CGC GCG GCG GGC GGG GCG GGG GCA CGG GGG G 3'

These sequences have been deposited in the GenBank database: *Weissella* sp, MF148162, MF148163, MF148164, MF148165, MF148166, MF148167, MF148168, MF148169, MF148170, MF148171, MF148172, MF148173; *Leuconostoc* sp. MF148174, MF148175, MF148176, MF148177, MF148178, MF148179; *Staphylococcus* sp. MF148180, MF148181, MF148182, MF148183; *Lactococcus* sp. MF148184, MF148185, MF148186, MF148187; MF148195, MF148196, MF148197, MF148198, MF148199, MF148200 *Streptococcus* sp. MF148188, MF148189, MF148190, MF148191, MF148192, MF148193, MF148194; *Lactococcus* sp. rumen bacterium clone, MF148201, MF148202, MF148203, MF148204; uncultured bacterial clone, MF148205, MF148206, MF148207, MF148208, MF148209, MF148210, MF148211, MF148212, MF148213, MF148214, MF148215, MF148216, MF148217, MF148218, MF148219, MF148220, MF148221, MF148222, MF148223, MF148224, MF148225, MF148226, MF148227. The initial taxonomic identification of ~1400bp bacterial 16S rRNA sequences (amplified by 27f and 1492r) is shown in Table 3.

**Table 3:** Summary of initial phylogenetic affiliations of bacterial sequences obtained from *H*. *contortus* adult worms, L3, eggs extracted from faeces (HEF) and laid *in vitro* (HEM). ~1400bp and ~1000bp sequences were amplified using universal and Firmicutes-specific primers respectively (URB- uncultured rumen bacteria). The phylogenetic affiliations were obtained by comparing bacterial sequences from *H*. *contortus* with those in the GenBank database using the BLASTn option in the NCBI website.

Bacteria					Adult worms		L3		HEF		HEM	
Phylum	Class	Order	Family	Genus	Sequence size (bp)							
					~1400	~1000bp	~1400	~1000	~1400	~1000	~1400	~1000
**Firmicutes**	Bacilli	Lactobacillales	Streptococcaceae	*Lactococcus*	8				2		2	
				*Streptococcus*	3	1	1	1	1	7		
			Leuconostocaceae	*Leuconostoc*	2	5	1	2		1	3	6
				*Weissella*	3	2	4	2		4	5	6
			Lactobacillaceae	*Lactobacillus*				1				
		Bacillales	Staphylococcaceae	*Staphylococcus*							4	
	Clostridia	Clostridiales	Clostridiaceae	*Clostridium* III	2				3			
			Clostridiaceae	*Clostridium* XI					12			
			Veillonellaceae	*Veillonella*							1	
			Catabacteriaceae	*Catabacter*	2							
			Lachnospiraceae	*Butyrivibrio*	5				2			
**Total**					**25**	**8**	**6**	**6**	**20**	**12**	**15**	**12**
**Tenericutes**	Mollicutes	Entomoplasmatales	Spiroplasmataceae	*Spiroplasma*					1			
**Total**					**0**	**0**	**0**	**0**	**1**	**0**	**0**	**0**
**Proteobacteria**	Alphaproteobacteria	Rhizobiales	Phyllobacteriaceae	*Mesorhizobium*	15		15					
			Rhizobiaceae	*Rhizobium*	3							
			Hyphomicrobiaceae	*Devosia*			1					
	Betaproteobacteria	Burkholderiales	Alcaligenaceae	*Achromobacter*			1					
				*Bordetella*			1					
				*Pusillimonas*			1					
			Comamonadaceae	*Acidovorax*							1	
				*Comamonas*			5					
				*Delftia*	1							
	Gammaproteobacteria	Enterobacteriales	Enterobacteriaceae	*Escherichia*			1		2		6	
		Pseudomonadales	Pseudomonadaceae	*Pseudomonas*	2				8			
		Xanthomonadales	Xanthomonadaceae	*Stenotrophomonas*	2				2		12	
**Total**					**23**	**0**	**25**		**12**		**19**	
**Bacteroidetes**	Bacteroidetes	Bacteroidales	Prevotellaceae	*Prevotella*	1							
				URB	7							
**Total**					**8**	**0**	**0**	**0**	**0**	**0**	**0**	**0**
**Total Sequences**					**57**	**8**	**31**	**6**	**32**	**12**	**35**	**12**

Sequences belonging to the phylum Bacteroidetes were identified only in adult worms and the majority were identified as uncultured rumen bacteria. Only a single *Spiroplasma* sequence was identified, in eggs collected from faeces.

Amongst the phylum Proteobacteria, the dominant genera were *Mesorhizobium*, *Stenotrophomonas*, *Pseudomonas*, *Comamonas* and *Rhizobium* and no sequence was common to adult worms, L3 and eggs. *Mesorhizobium* were found only in adult worms and L3, the majority of the *Stenotrophomonas* in eggs laid *in vitro* and *Pseudomonas* in eggs collected from faeces. As Proteobacteria are ubiquitous in the environment [52], they may be acquired on to the surface of parasites from their environment, whereas *Mesorhizobium*, *Stenotrophomonas* and *Pseudomonas* may be gut residents. Sequences of the phylum Proteobacteria were not subjected to detailed analysis because of the high sequence variation identified by the universal bacterial 16S rRNA primer pair. Further phylogenetic analysis was carried out only for phylum Firmicutes sequences, which were the only ones identified in all three *H*. *contortus* life-cycle stages (adult worms, L3 and eggs).

### Phylogenetic analysis of phylum Firmicutes ~1400bp and ~1100bp 16S rRNA sequences

**Fig 2:**
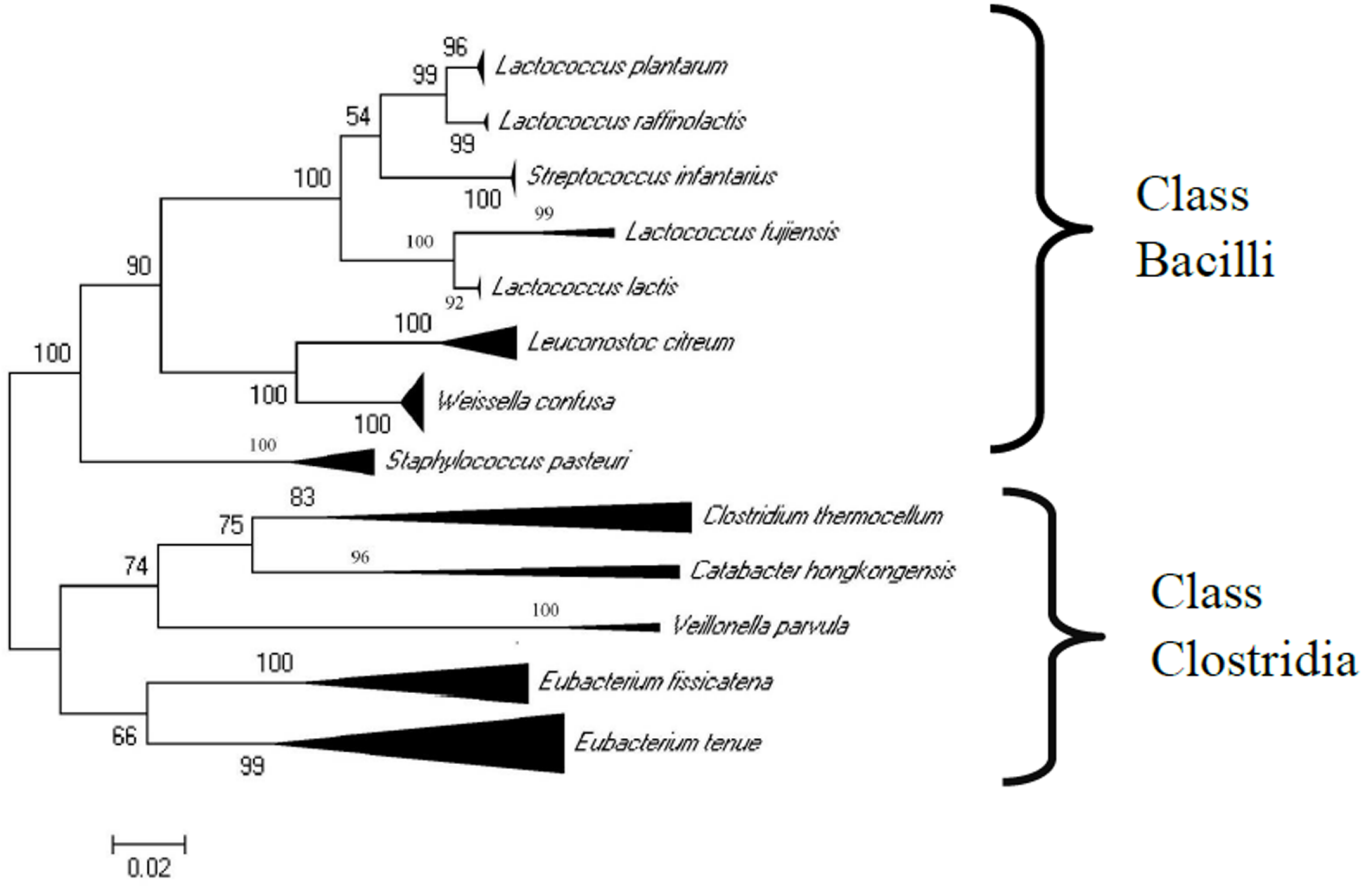
Phylogenetic tree (based upon the Maximum Likelihood method) of phylum Firmicutes 16S rRNA gene sequences from *H*. *contortus* using the universal primer set 27f and 1492r and reference 16S rRNA gene sequences. The 13 groups were identified from closest type strain sequences. Sequences which have been compressed are represented as triangles. Bootstrap values are shown at each node (percent of 500 replicates). The scale bar indicates 0.02 nucleotide substitutions per nucleotide position.

Phylum Firmicutes sequences had the same phylogenetic affiliations and the phylogenetic trees had similar topology and robustness for the Maximum Likelihood (ML), Neighbour Joining (NJ) and Maximum Parsimony methods. Fig 2 shows the tree generated by the ML method for the ~ 1400bp sequences, and detailed trees for ~ 1400bp and ~ 1000bp sequences are shown in S4–S6 Figs. Sequences grouped into 13 clusters in the phylogenetic analysis of ~ 1400bp sequences (using the universal primer set 27f and 1492r) (Table 4) and 4 clusters in the analysis of ~1000bp sequences (using the Firmicutes-specific set 27f and 1040firmR) (Table 5).

**Table 4:** Taxonomic assignment of ~1400bp 16S rRNA sequences from *H*. *contortus*, based on the closest cultured and type strain relatives. Sequence similarities were calculated from aligned gene sequences using the Geneious software package and MEGA 5.0. HA: adult worms; HL: L3; HEM: eggs laid *in vitro*; HEF: eggs collected from faeces. Type strains are designated ^T^.

Cluster	Bacterial sequences from *H*. *contortus*	Genus	Closest cultured and type strain (GenBank accession)	Similarity (%)
CLP	HA2, HA8, HA20, HA25, HA719	*Lactococcus*	*Lactococcus plantarum* DSM 20686^T^ (EF694029)	99.7
	HEM519			98.6
CLR	HA3, HA819	*Lactococcus*	*Lactococcus raffinolactis* DSM 20443^T^ (NR_044359)	99.9
CSI	HA1, HA18, HA9, HL11, HEF723	*Streptococcus*	*Streptococcus infantarius* subsp. *coli* NCDO964^T^ (AF429763)	99.6–99.7
CL	HEF1	*Lactococcus*	*Lactococcus lactis* subsp. *lactis* NCDO_604^T^ (DSM 20481) (AB100803))	93.5
			*Lactococcus fujiensis* strain: NJ317 (AB485959	94.1
CLL	HA14, HEF8, HEM24	*Lactococcus*	*Lactococcus lactis* subsp. *lactis* NCDO_604^T^ (DSM 20481) (AB100803)	99.7–100
CLC	HA17, HA219, HEM23, HEM819	*Leuconostoc*	*Leuconostoc citreum* ATCC 49370^T^ (AF111948)	99.5–99.8
			*Leuconostoc citreum* strain B/ 110-1-2 (FJ716698)	99.4–99.8
	HL28			96.8; 96.6
	HEM619			92;92
CWC	HA5, HA10, HA12, HEM20-22, HEM919, HEM1019, HL8, HL23, HL32	*Weissella*	*Weissella confusa* JCM1093^T^ (AB023241)	99.6–100
	HL12			96.8
CSP	HEM29, HEM319, HEM419	*Staphylococcus*	*Staphylococcus pasteuri* ATCC51129^T^ (AB009944)	99.1–99.7
	HEM219			95.6
CCT*	HA15-16, HEF1123, HEF1822, HEF2022	*Clostridium* III *Catabacter*	*Clostridium thermocellum* DSM 1237 ^T^ (L09173)	79.9–81.3
			*Clostridium sufflavum* strain CDT-1 (NR_041497)	80.9–82.2
CCH*	HA119, HA19		*Catabacter hongkongensis* strain: JCM 17853^T^ (AB671763)	83.5–85.4
CVP	HEM25	*Veillonella*	*Veillonella parvula* ATCC 10790^T^ (AY995767)	91.2
CET	HEF223, HEF323, HEF423, HEF523, HEF823, HEF923, HEF1023, HEF1323, HEF1523, HEF1922, HEF2222	*Clostridium* XI	*Eubacterium tenue* DSM 20695^T^ (FR749984)	93.8–98
			*Clostridium sordellii* strain HT5 (DQ978216)	94.3–98.2
	HEF623			83.8; 83.8
CEF*	HA4, HA6, HA11, HA13, HA21, HEF1423 and HEF1623	*Eubacterium Robinsoniella*	*Eubacterium fissicatena* DSM:3598^T^ (GU985201)	85.5–91.6
			*Robinsoniella peoriensis* strain PPC44 (AF445283)	84.9–92

*Sequences belonged to these clusters had very low sequences similarity with known cultured and type stains sequences but always fall into stable clusters in phylogenetic analysis based upon the maximum likelihood and neighbour joining methods.

**Table 5:** Taxonomic assignment of ~ 1000bp phylum Firmicutes 16S rRNA sequences from *H*. *contortus*, based on the closest cultured and type strain relatives, identified by comparative analysis of 16S rRNA gene sequences. Sequence similarities were calculated from aligned gene sequences in Geneious software package and MEGA 5.0 (A: adult worms; L: L3; ME: eggs laid *in vitro* and FE: eggs collected from faeces). Type strains are designated ^T^.

Cluster	Bacterial sequences from *H*. *contortus*	Genus	Closest cultured and type strain (GenBank accession)	Similarity (%)
CFLC	A2, A4-6, A9, L4-5, FE2, ME2-5, ME7-8	*Leuconostoc*	*Leuconostoc citreum strain* ATCC49370^T^ (NR_041727)	>99
CFWC	A3, A8, L1, L6, FE1, FE3, FE7, FE11, ME1, ME6, ME9-12	*Weissella*	*Weissella confusa gene* JCM1093^T^ (AB596944)	>99
CFLF	L3	*Lactobacillus*	*Lactobacillus ingluviei* strain KR3^T^ (NR028810)	94.5–95.3
			*Lactobacillus fermentum* strain KN02 (HQ650232)	>99
CFSE	A1, L2, FE4-6, FE8-10, FE12	*Streptococcus*	*Streptococcus infantarius* strain HDP90104^T^ (NR_028761)	97.3–97.9
			*Streptococcus equinus* strain: BP1-7 (AB563264)	>99

Detailed phylogenetic analysis of the relatively small number of sequences of the order Clostridiales suggested that many may belong to yet to be described genera or species, as these sequences had very low sequence similarity with type strain sequences and their groups also had low bootstrap values. Most of the clone sequences within the CCT cluster grouped separately from the known type strain *Clostridium thermocellum* and the cultured species *Clostridium sufflavum*. These clone sequences had a low bootstrap value (55) with their type strain and cultured strain sequences in the detailed phylogenetic tree (S5 Fig). Sequences belonging to the genera *Clostridium* and *Lactococcus* were present in adult worms and faecal eggs, but no sequences belonging to *Clostridium* sp. were identified in L3, probably due to their removal by exsheathing. *Clostridium* sp. are mammalian gut residents [43–46], suggesting sheep gut bacteria are present on the surface of the parasites or in the worm gut.

Sequences (~1400bp) belonging to the lactic acid bacteria *Lactococcus*, *Streptococcus*, *Leuconostoc* and *Weissella* were identified in adult worms, L3 and eggs, similar to the findings from the short sequences retrieved from DGGE bands (Table 2). These sequences had over 99% sequence similarity with those from type strains identified as *Weissella confusa*, *Leuconostoc citreum*, *Lactococcus plantarum*, *Lactococcus raffinolactis*, *Lactococcus lactis*, and *Streptococcus infantarius*. Although sequences (~1400bp) from the genera *Weissella* and *Leuconostoc* were not found in eggs collected from faeces, partial 16S rRNA bacterial sequences (~1000bp) using the phylum Firmicutes-specific primer confirmed that *Weissella* and *Leuconostoc* sequences can be obtained from all life-cycle stages, including eggs collected from faeces. Notably, those sequences amplified from *in vitro* laid eggs using the phylum Fimicutes-specific primer were all *Leuconostoc* or *Weissella*. The failure to find lactic acid bacterial sequences from faecal eggs is not surprising, as most belonged to *Clostridium* sp. and the most abundant species would dominate the clones used for sequencing. The reason for the large number of sequences of *Clostridium* sp. could be either primer bias or the presence of large numbers faecal bacteria adhering to the eggs.

### Location of bacteria in *H*. *contortus*

**Fig 3:**
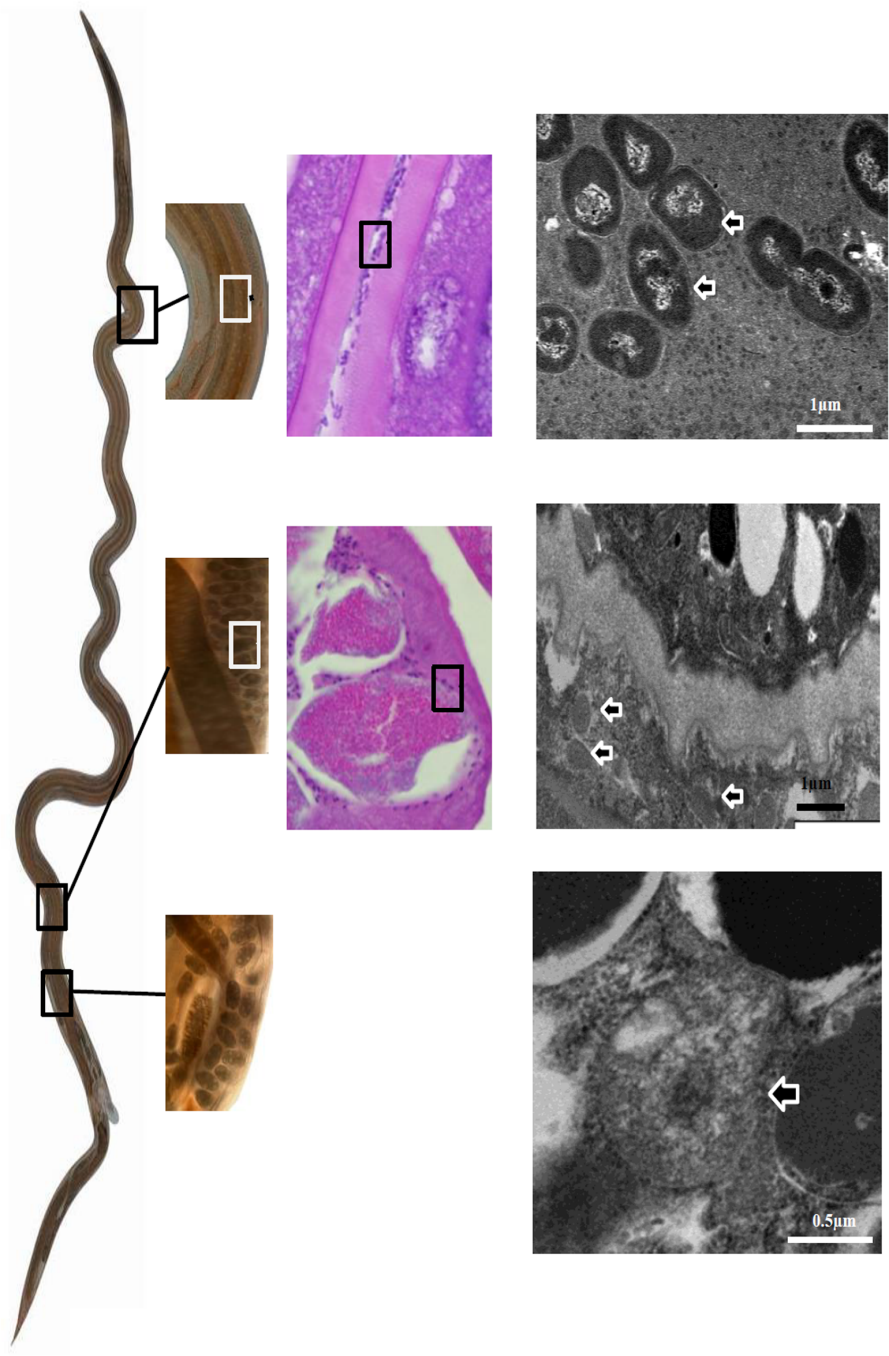
Location and morphology of bacteria in the gut (top), the uterus (centre) and eggs (bottom) of an adult female *H*. *contortus*. Left: LM image of a whole unstained worm showing the sites of collection of tissues; middle: LM images of H & E stained tissues; right: TEM images. Bacteria are shown at successively higher magnifications. Those in the eggs were not seen in LM sections. In H & E stained sections, bacteria are shown in boxes and in TEM sections are indicated by arrows.

Symbionts were identified by a combination of light microscopy (LM), transmission electron microscopy (TEM) and fluorescence in situ hybridisation (FISH). Bacteria were apparent in three locations in histological sections of adult worms: in the gut lumen of male and female worms, and in females also within the uterus and eggs. The group-, class- and species-specific FISH probes (Table 2) used to identify the morphology and locations of bacteria in *H*. *contortus* were selected based on the phylogenetic analyses. The bacteria visible in a female worm by LM and TEM are summarised in Fig 3.

Bacteria could not be seen on the surface of the worms, using either LM or FISH. Live helminths actively remove cells [53,54], chemicals [55], antibodies [55–57] and lectins [54,58] attached to their surface by continuously replacing their cuticles. Therefore, it is unlikely that there are permanent bacterial communities on the surface of *H*. *contortus*, whereas some free living marine nematodes carry large permanent populations of sulphur-oxidising ectosymbionts which provide nutrients to their host [4,59].

#### Gut bacteria

Bacteria in the gut lumen of female worms in sections stained with H & E were a mixed population of gram-positive and gram-negative bacteria. In TEM sections, there were diverse morphotypes in the gut in a single worm and these differed amongst worms, although many worm sections contained no visible bacteria. Bacteria were seen in the gut lumen and none was attached to microvilli or within any specialised structures. Gut bacteria in adult worms hybridised with the eubacterial probe (EUB338) and in a few sections also with the Strc493 probe, which hybridises with most *Streptococcus* sp. and some *Lactococcus* sp. Neither the lactic acid bacterial group- nor the *Weissella* species-specific probes targeted any bacteria in the gut. Although Proteobacterial sequences were identified in clone libraries, these bacteria were not visualised using class-specific Proteobacterial FISH probes.

The *H*. *contortus* microbiota was mainly comprised of members of the phyla *Proteobacteria*, *Firmicutes* and *Bacteroidetes* (Tables 1 and 3). A large proportion of sequences were consistent with the vertically-transmitted *Weissella*/*Leuconostoc* endosymbionts and the *Lactococcus/Streptococcus* sp. seen in the uterus of female worms. The remainder, which are likely to be attached to the cuticle or in transit or resident in the intestine of L3 or adult worms, were dominated by Proteobacteria, *Clostridium* sp. and *Lactococcus* sp. Sequences belonging to Bacteroidetes, typical ruminant foregut bacteria [60–63], were identified in adult worms, but not L3 or eggs (Fig 1 and Table 1). Rumen bacteria can remain alive in abomasal contents, particularly when the pH is raised by the presence of abomasal parasites [64] and either dead or live rumen bacterial cells could contribute to the DNA and the sequences subsequently identified in the adult worm samples. In adult worms and L3, the most frequently identified 16S rRNA sequences belonging to the phylum Proteobacteria were from *Mesorhizobium* sp., which are agriculturally important soil and rhizosphere bacteria [65]. Some of these may have been become associated with the free living stages (L1 or L2) of the parasite, which feed on bacteria, and remained in the dormant, non-feeding L3 stage and subsequently survived in L4 and adult worms. Proteobacteria also dominated the microbiomes of the plant-parasitic nematode *Meloidogyne incognita* [50] and free-living species [13–15].

The gut in many of the worm sections contained no bacteria, which could either be the result of the delay in recovery using the agar method and preparation for FISH or from expulsion of bacteria by agar taken in through the mouth and moving along the intestine. Although not detected by FISH probes, even a small amount of DNA from these bacteria could have been detected by PCR, especially if the primer pair preferentially amplified bacterial sequences from the phylum Proteobacteria over the phylum Firmicutes. The rapid transit time of contents through the nematode gut, estimated to be less than 2 min in *C*. *elegans* [66] suggests than for a comprehensive study of the *H*. *contortus* gut microbiome, the worms should be manually collected from digesta and externally cleaned very rapidly to prevent loss of gut contents.

#### Bacteria in the uterus

In most female worms, there were numerous gram-positive bacteria in the distal uterus, but not in the proximal uterus near the ovaries. In TEM sections, they were densely grouped near the wall of the uterus between the wall and the egg shells. Bacteria were of a single morphotype, 300–500 nm in diameter, smaller than those in either the eggs or in the gut, but none had a thick cell wall characteristic of gram-positive bacteria. Sperm were also present within the uterus adjacent to fully formed eggs. These coccoid or diplococcoid bacteria are close relatives of either *Lactococcus* sp. or *Streptococcus* sp., as they were targeted by the Strc493 FISH probe. The Strc493 probe was not able to be combined with other species-specific probes because of different optimal hybridisation stringency conditions. The bacteria detected in the uterus were not the same as those present in eggs, as in separate FISH experiments, none of the probes Lab158, Wgp and S-G-Wei-0121-a-S-20 hybridised with bacteria within the uterus, unlike those in eggs.

Despite their large numbers, these bacteria appeared not to be pathogens for the nematodes, which were the usual size for the species, fully active and contained eggs of normal morphology. This nematode culture is fully pathogenic to sheep and has the pre-patent period and high egg production usual for *H*. *contortus*. The possible routes of entry of those bacteria into the uterus could either from the environment (abomasum) or transmitted by male worms during mating, after which they become resident in the uterus. The worms could be bacterial vectors, as some *Streptococcus* sp. are opportunistic pathogens, while others are commensal bacteria in animals and humans [67]. Their location in the distal, rather than proximal, uterus suggests they are acquired at each generation and not vertically transmitted.

#### Bacteria in eggs

**Fig 4:**
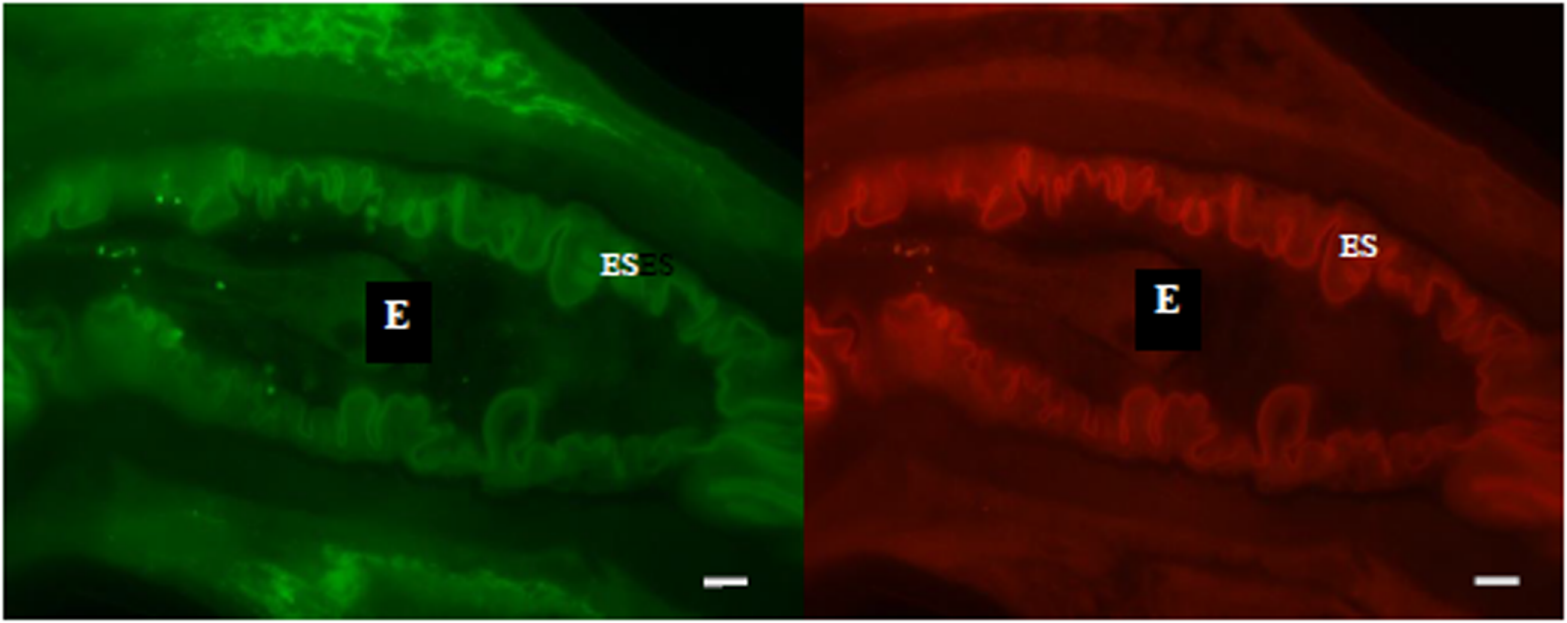
Bacteria inside an egg, near the ovipositor in a female worm. Bacteria were targeted by EUB338 (FITC-labelled, left) and Wgp (Cy3-labelled, right) probes. The EUB338 probe targets all bacteria and the Wgp probe targets *Weissella* sp. Not all bacteria were targeted by the Wgp probe. E: egg; ES: egg shell. Bar: 10μm.

Eggs collected from faeces and also in female worms contained a small number of spherical bacteria, 800–1000 nm in diameter, which were very close relatives of *W*. *confusa*. They were clearly seen within eggs in 2 of 28 TEM sections of female worms, but could not be recognised in LM sections. Although clearly defined, the cell walls were not as thick as those in bacteria in other locations. Bacteria within eggs in females were hybridised by the lactic acid bacterial group-specific probe Lab158, as well as the *Weissella* species-specific probes (Wgp and S-G-Wei-0121-a-S-20) (Fig 4). Lab158 also hybridised with eggs in faeces. There may be closely related lactic acid bacteria in the eggs, as not all could be targeted by *Weissella* sp-specific probes, because there were more EUB338 signals than that from Wgp and S-G-Wei-0121-a-S-20. The bacteria were either coccoid or diplococcoid, consistent with TEM images, and scattered throughout the *H*. *contortus* egg as individual cells or in small clusters when viewed at different focal planes in confocal microscopic images.

Other bacterial sequences were also present in clone libraries constructed from eggs, although no bacteria were seen either by FISH or TEM on the egg surface. This is consistent with RNA/DNA of faecal bacteria contaminating eggs in faeces or carried by female worms attached to eggs laid *in vitro*. *Clostridium* sp. are mammalian gut residents [43–46] and sequences belonging to this genus were prominent in eggs collected from faeces. Similarly, sequences belonging to *Sternotrophomonas* sp. were dominant in the clone library of eggs laid *in vitro*.

Maternal transmission of the *Weissella/Leuconostoc* endosymbionts is strongly supported by their visualisation in eggs, both in the female and after laying, and identification in all three life-cycle stages of nearly full length (~1400bp and ~1000bp) 16S rRNA gene sequences and short sequences (~190bp) from DGGE bands. Despite identifying similar bacterial sequences in L3 and male and female worms, these bacteria were not able to be visualised in L3 and male worms by FISH, perhaps due to dormancy of the bacteria. In L3 sections, background fluorescence was so strong that true FISH signals could not be distinguished from false positives. Therefore, the location of bacteria in sections of L3 and male worms and the details of the route of vertical transmission remain unknown.

The most likely source of the endosymbionts is rumen fluid, in which they form a minor, but variable, component of the microbiome [68], however, they also have been detected in plants, fermented foods, meat products and in human and animal gut contents, milk and saliva [69]. *Weissella* are also rare opportunistic pathogens of humans and animals causing infections of the heart and artificial joints, abscesses and bacteraemia, probably after entry from the intestinal mucosa [70,71]. They do not appear to be pathogenic for *H*. *contortus*, although experimental feeding to *C*. *elegans* caused a moderately extended life span, compared with worms fed *Escherichia coli*, due to dietary restriction and stress induction [72]. *Weissella* sp. may use *H*. *contortus* as a vector, similar to the situation involving vertical transmission of endosymbiotic *Neorickettsia* in parasitic trematodes (flatworms) and horizontal transmission to trematode vertebrate hosts, which then develop serious diseases [73–75].

The *Weissella*/*Leuconostoc* endosymbionts in *H*. *contortus* could be evolving from free-living to mutualistic endosymbiotic bacteria. This process has been induced experimentally in Rhizobia in legumes through acquiring essential genes and genome re-modelling [76–78], leading ultimately to a reduced symbiotic genome as non-essential genes are lost [3,79]. Horizontal transfer of genes from associated bacteria or spontaneous mutations similarly may be shaping the development of free-living *Weissella* into endosymbionts of *H*. *contortus*. They may be at the transitional stage between free-living and endosymbionts, with gene sequences evolving in only some of the bacteria in the population; this could explain why fewer symbionts in eggs were targeted by *Weissella* species-specific probes than by the Lab158 FISH probe, resulting in an erroneously interpretation of multiple species. The specific probes may hybridise with the highly variable regions, which may be evolving, whereas Lab158 targets a conserved region in the 16S rRNA gene of most of the lactic acid bacterial group; this region may not be evolving at this time.

The best known vertically transmitted symbionts are the *Wolbachia* and Verrucomicrobia, which manipulate respectively the biology of filarial [23,29] and plant parasitic nematodes [21,22]. More recently, a *Comomonas* sp. has identified in eggs, the gut cells of L3 and adult *Spirocerca lupi*, a parasite principally of canids [80]. Although this symbiont was present in the strain of *S*. *lupi* prevalent in Israel, it did not appear to be in the parasites in South Africa [81]. A similar situation could exist in *H*. *contortus*, in which the symbiont is currently known only in a laboratory strain of the nematode and its distribution in the field is unknown.

### Conclusions

The microbial communities of *H*. *contortus* were shown by PCR-DGGE and constructing clone libraries of sequences to differ from the communities in the natural environments of adult parasites in the abomasum or developing L3 in faeces. Detailed phylogenetic evolutionary relationships showed that members of the phyla Proteobacteria, Firmicutes and Bacteroidetes were associated with adult worms, larvae and eggs in faeces and laid *in vitro*. Bacteria were identified in eggs, the female reproductive tract and the gut of adult and L3 larvae using the universal FISH Eub338 probe, which targets most bacteria, and were also seen by light and transmission electron microscopy. Those in the reproductive tract were of two different morphotypes and their sequences matched to unrelated type species in the phylum Firmicutes. *Streptococcus* or *Lactococcus* sp. were targeted within the distal uterus with the probe Strc493, whereas sequences from the genera *Weissella* and *Leuconostoc* were found in all life-cycle stages, except eggs collected from faeces, which were dominated by sequences belonging to *Clostridium* sp. Bacteria closely related to *W*. *confusa* were identified both by PCR-DGGE short sequences and in clone libraries of nearly full length 16S rRNA bacterial sequences in all life-cycle stages; they were subsequently visualised only in eggs by fluorescent *in situ* hybridisation (FISH) with group-specific probes despite detection of their DNA in L3, female and male worms. This strongly suggests they are vertically transmitted endosymbionts of a laboratory strain of *H*. *contortus*.

## Materials and methods

### Parasites

Maintenance in the laboratory for 10 years of a pure culture of *H*. *contortus*, originally collected from the field, by regular passage through sheep and recovery of adult worms from euthanased sheep were carried out in accordance with the requirements of the Massey University Animal Ethics Committee approval #09/11 for this project.

Abomasal fluid and adult worms were recovered from infected sheep on Day 21 post infection [82]. Briefly, the abomasum was removed and abomasal contents and saline washings were mixed 2:1 with warmed 3% agar, allowed to set and the solidified blocks incubated at 37°C in a saline bath. Clumps of parasites were collected from the saline soon after emergence and placed in medium appropriate for microscopy or molecular biology. Some samples of adult worms were also manually collected from abomasal contents. Male and female populations were separated under a dissecting microscope, based on morphological differences. Worms were placed on a sterile filter and washed alternately five times with 4% sodium hypochlorite for 5–10 sec and rinsed with approximately 200 ml ultrapure water. Ultrapure water was prepared from MilliQ water by filter sterilisation, autoclaving and ultraviolet irradiation for 48 h.

Approximately 500–600 eggs were collected either from faeces or after laying by female worms *in vitro*. After recovery from agar, 15 adult females/tube were placed in 1.5 ml phosphate buffered saline (PBS) in each microcentrifuge tube to lay eggs during an overnight incubation at 37°C. After manual removal of the worms, the egg suspensions were pooled and the eggs separated and washed on a sterile filter, as described above for adult worms. Eggs were separated from faeces by sequentially passing through sieves down to 20 μm mesh, which retained the eggs along with some particulate matter. This suspension was placed on top of a saturated NaCl solution for 5 min and eggs allowed to stick to a glass surface placed on top. The eggs were washed on to a sterile filter and treated as described above for laid eggs *in vitro*.

L3 were cultured in faeces collected into faecal bags on infected sheep. Faeces were placed in trays, moistened, covered and cultured for 10 days at 22–24°C. L3 were separated from faeces by their movement through paper tissues (Baermannisation), washed and stored in reverse osmosis (RO) water at 10°C. Before use, L3 were exsheathed by incubating at 37°C in 0.05% sodium hypochlorite solution (Clark Product Ltd, Rotorua, New Zealand) for 15–20 min. After confirming microscopically that 95–100% had exsheathed, they were again Baermannised in ultrapure water for 12 h.

### Molecular fingerprinting of bacterial communities in *H*. *contortus*

DNA was extracted from parasites and samples from their environment, bacterial 16S rRNA genes were amplified and bacterial sequences were subjected to phylogenetic analysis.

### Extraction of DNA

DNA was extracted from 0.5 g samples of 4% sodium hypochlorite washed adult worms, exsheathed L3 and eggs, as well as abomasal fluid and faeces. Samples were homogenised using a sterile micro-centrifuge pestle, incubated in lysis solution, plus 20 μl of 20 mg/ml proteinase K (Life Technologies) solution for 2.5 h at 37°C. DNA was extracted from 200 μl samples, as described in the manufacturer’s manual of the QIAamp DNA stool-kit (Qiagen, Hilden, Germany). The quantity and the purity of the DNA were determined using a NanoDrop ND-1000 UV-Vis spectrophotometer (NanoDrop Technologies) or using the Qubit ™ ds DNA HS Assay Kits on a Qubit fluorometer (Invitrogen) according to the manufacturer’s instructions. Samples were stored at -20°C for downstream applications.

#### PCR-DGGE

Bacterial 16S rRNA genes were amplified in a 50 μl reaction mixture containing: 5.1 μl 10X PCR buffer with Mg^2+^, 5 μl of 2 mM dNTP, 0.5 μl of 10 μM forward primer and 0.5 μl of 10 μM reverse primer, 2.5 U (0.5 μl) of native Taq DNA polymerase (Roche Diagnostics, Mannheim, Germany), 5 μl of template and 33.4 μl of ultrapure water. The following touchdown PCR was used: stage I: 3 min initial denaturation at 95°C, followed by stage II: 10 cycles of denaturation (30 sec at 95°C), initial annealing (30 sec at 62°C) and elongation (30 sec at 72°C), -0.5°C/cycle, followed by stage III: 26 cycles of denaturation (30 sec at 95°C), annealing (30 sec 57°C) and elongation (30 sec at 72°C) with final elongation at 72°C for 10 min. PCR amplifications were carried out with the universal bacterial primers, 338f and 518r (Table 1); the forward primer (338f) had a 40-nucleotide GC-clamp added to the 5’-end.

Amplified DNA was analysed by agarose gel electrophoresis. Gels were stained with SYBR safe DNA stain (Invitrogen), visualised using UV trans-illumination and photographed using a BIO-RAD Molecular Imager^®^ Gel Doc™ XR (BIO-RAD Laboratories, Milan, Italy). The concentration of the extracted DNA was measured and the purity was determined using a NanoDrop ND-1000 UV-Vis spectrophotometer (NanoDrop Technologies, Wilmington, DE, USA) according to the manufacturer’s instructions. PCR products were purified with the Wizard^®^ SV Gel and PCR Clean-Up System (Promega, Madison, WI, USA).

Purified PCR products (300 ng) were mixed with equal volumes of DGGE loading dye (0.05% [w/v] bromophenol blue, 0.05% [w/v] xylene cyanol, 70% [w/v] glycerol in water, pH 8.0) and loaded into the DGGE gel wells. A 1 kb-plus marker (Invitrogen) was also run on each gel. The gels were electrophoresed with 1X TAE buffer which contained 40 mM tris (hydroxymethyl) aminomethane, 65 mM acetic acid and 10 mM EDTA, adjusted to pH 8 with 5 M NaOH. The electrophoresis was performed at 60°C for 5 h at 200 V. After the electrophoresis, gels were stained with 3 μl of SYBR Gold (Invitrogen) in 600 ml of MQ water for 20 min on a shaker and then destained overnight. The gel was visualised using UV trans-illumination and photographed.

### Sequences from DGGE bands

Bands of interest in adult, L3 and egg samples were excised and aseptically transferred into sterile centrifuge tubes. 100 μl ultrapure water was added to the tube containing the excised band and vortexed for 1min, then the water was removed. Gel slices were finely broken in 50 μl of ultrapure water using sterile pipette tips. Tubes were incubated overnight at 4°C and the following day were vortexed for 5 sec and centrifuged at 28,000 *g* for 1 min. The supernatants were transferred to sterile centrifuge tubes and the DNA fragments in the supernatant were re-amplified with the primers 338f and 518r, using the touchdown PCR protocol with an initial annealing temperature of 62°C. Purified PCR products were cloned by ligation into a plasmid vector (pCR 2.1TOPO-TA cloning vector, Invitrogen) and transformed into chemically competent *E*.*coli* TOP-10 cells, using a TOPO-TA cloning system (Invitrogen) according to the manufacturer’s instructions. Amplified PCR products were sequenced using the primer M13f by Macrogen Inc. (Seoul, Republic of Korea).

Vector sequences were removed using MEGA version 5 (Molecular Evolutionary Genetics Analysis) [83]. The sequences were checked for quality of the chromatogram (evenly-spaced peaks) and miscalled nucleotides. Good quality sequences were analysed with known sequences available in the GenBank database. The BLASTn search option of the National Center for Biotechnology Information (NCBI) web site (http://www.ncbi.nlm.nih.gov) was used to compare sequences of close evolutionary relatives with sequences obtained from the DGGE bands [84].

### Phylogenetic analysis of bacterial sequences from adult worms, L3 and eggs

Nearly complete length (~1400bp) and ~1000bp 16S rRNA gene sequences were amplified respectively with the universal bacterial primer set 27f and 1492r or 27f and the phylum Firmicutes-specific primer 1040firmR (Table 1). The PCR mixture (25 μl) contained 2.6 μl of 10X reaction buffer with MgCl_2_, 2.5 μl of dNTP, 0.5 μl of 0.5 μM of each primer, 0.5 μl of 2.5 U of Taq DNA polymerase (Roche), and 15.9 μl DNA-free ultrapure water. The PCR was carried out using the touchdown PCR programme with an initial annealing temperature of 62°C.

For the detailed phylogenetic analysis, the ~1400bp and ~1000bp bacterial sequences were matched with those in the GenBank database [85] using the BLASTn option [84] to obtain the closest sequences of uncultured bacteria, cultured isolates and type strains. Additionally, the sequence match option of Ribosomal Database Project (RDP) was used to obtain information on the closest type strain. For phylogenetic tree building, sequences from the GenBank database from uncultured bacteria with similarity >99%, the closest cultured isolate and type strain sequences which had the highest similarity to the sequence were combined with those from *H*. *contortus*. Sequences were globally aligned with ClustalW using the MEGA 5.0 software package [83]. The alignment was manually corrected by comparison with previously identified conserved regions.

The online chimeric detection programme Bellerophon [86] (Huber et al., 2004) was used to identify chimeric sequences. The phylogenetic affiliations of sequences were created with the phylogeny option of MEGA 5.0 [83] (Tamura et al., 2011), using the default settings for the NJ, ML and parsimony methods. Phylogenetic trees of ~1400bp sequences obtained from NJ, ML and parsimony were compared with each other to verify the robustness of tree topology. Additionally, two NJ trees were created from the first and last ~400bp of aligned ~1400bp sequences and compared with each other for further detection of chimeric sequences. Potential chimeric sequences were excluded from further tree building analysis. Distance matrices of aligned sequences were also made using the Geneious software package [87]. The same procedure was carried out for ~1000bp sequences. The final dendrograms of sequences of phylum Firmicutes (~1400bp and ~1000bp) were inferred using the NJ, ML and parsimony methods. Each analysis included sequences from the closest cultured and type strains and bacterial sequences identified in *H*. *contortus*.

### Microscopy

#### Light microscopy (LM)

Adult worms were fixed in 10% [v/v] neutral buffered formalin overnight, then routinely processed in a Leica TP1050 Tissue processor (Global Science and Technology, Auckland, New Zealand) and paraffin embedded (Leica Histo Embedder, Germany). Sections 5 μm thick were cut on a Leica RM 2235 manual rotary ultramicrotome (Wetzlar, Germany), using a S35 Feather disposable microtome blade (Osaka, Japan). Sections were de-waxed and stained with hematoxylin and eosin (H & E) using a Leica Autostainer XL (Global Science and Technology, Auckland, New Zealand) or by the Gram Twort method. Slides were washed, dried and a drop of Entellan immersion oil (Merck New Zealand Ltd, Auckland, and NZ) was added as a mounting solution. Sections were covered with cover slips and viewed under with an OLYMPUS BX61 microscope (OLYMPUS, Tokyo, Japan).

#### Transmission electron microscopy

Adult female worms were sliced into pieces 6–8 mm long, fixed for 2–3 days in 3% glutaraldehyde and 2% formaldehyde in 0.1 M phosphate buffer (Na_2_HPO_4_.12H_2_O and KH_2_PO_4_), pH 7.2 and post-fixed in 1% OsO_4_ in phosphate buffer. Tissues were embedded in resin and 1 μm thick sections cut on an Ultra-microtome (Leica Microsytems, Wetzlar, Germany). Sections were stained with 0.05% toluidine blue and areas of interest were chosen by LM. Sections 100 nm were cut and double stained with uranyl acetate and lead citrate and observed by electron microscopy (Philips CM10 Transmission Electron Microscope with SIS Morada high-resolution digital imaging) at 60 kV at the Manawatu Microscopy and Imaging Centre, Massey University (MMIC).

### Fluorescence in situ hybridisation (FISH)

Eggs from faeces and laid *in vitro*, exsheathed L3 and male and female worms were collected as described above. To reduce gut emptying, female worms were also collected from abomasal contents after euthanasia of the sheep and fixed immediately. Adult worms and L3 were straightened by incubating for approximately 12 h at 4°C in PBS, then all lifecycle stages were fixed overnight at 4°C in 4% paraformaldehyde in PBS (PFA). After residual PFA had been removed by washing twice with PBS, adult worms were transferred to 70% ethanol and stored at -20°C until automated, routine histological processing through graded alcohol solutions and 100% xylene (Leica TP1050 tissue processor, Wetzlar, Germany) and paraffin embedding (Leica Histo Embedder, Wetzlar, Germany). Eggs and L3 were processed in microcentrifuge tubes and centrifuged at 17,100 *g* for 1 min between dehydrating and washing steps. This was followed by serial immersion of the samples in ethanol-xylene solutions of 3:1, 1:1 and 1:3 [v/v] and finally in 100% xylene for 10 min. After removing the xylene, the samples were embedded in paraffin blocks (Leica Histo Embedder).

Sections 3 μm thick were cut as for LM and 2 sections, each containing 3 adult worms, 100–200 eggs or L3, were placed on each slide (Menzel-Glaser Superfrost, Lomb Scientific Pty Ltd, Sydney, Australia). Sections were de-parafinised by heating for 3–5 sec at 100°C, immersed in 100% xylene for 15 min and then in 100% ethanol for 15 min. These two steps were repeated twice and then the slides were washed in MQ water. The slides were thoroughly air-dried and treated with 1 mg/ml lysozyme (Sigma) for 10 min for the Lab158, Strc493, Wgp and S-G-Wei-0121-a-S-20 probes, but not Proteobacterial class-specific probes. Lysozyme was removed under running tap water and the slides were air-dried thoroughly.

The group-, class- and species-specific probes Lab158, Wgp, S-G-Wei-0121-a-S-20, Strc493, Alf73a, Beta1 and SteMal_439 were selected from the literature to target the bacterial species identified from ~190bp and ~1400bp sequences from *H*. *contortus* (Table 1) and analysed using the probeBase website [88] (Loy et al., 2007). In the absence of a suitable 16S rRNA probe for Alphaproteobacteria, a 23S rRNA targeted probe was selected. Labelled (Cy3 or FITC) probes were purchased from Eurofins MWG Operon (Ebersberg, Germany) and Sigma Aldrich (Auckland, New Zealand). The specificity was determined for 10 reference cultures (S1 Table) and the optimum formamide concentration and hybridisation stringency for each probe were determined (S2 Table) as the formamide concentration immediately below that in which specific signals decreased and there were no non-specific signals from non-target species.

FISH was carried at 46°C for 2 h in humidified containers on multiple slides, each containing two consecutive serial sections of either 3 adult worms, 100–200 eggs or 100–200 L3, isolated by drawing a hydrophobic barrier around each nematode section. Hybridisation buffer (20 mM tris-HCl, 0.9 M NaCl, 0.01% sodium dodecyl sulphate, pH 7.2), containing 50 ng/μl probe and formamide was added to cover each section on the slide. Each experiment included a control for autofluorescence (hybridisation buffer without probes) and a negative control (Non-EUB338 labelled with FITC or Cy3) and the universal bacterial EUB338. Following hybridisation, each slide was rinsed immediately using a pipette containing the appropriate washing buffer (pre-heated to 48°C) and then placed in a tube containing washing buffer at 48°C for 10–15 min. After removal from the washing buffer, the slides were immediately rinsed briefly in a beaker of ice-cold distilled water and then thoroughly air-dried.

Sections were mounted, viewed under both phase contrast and at appropriate wave lengths under a confocal laser scanning microscope (Leica TCS SP5 DM 6000B Leica Microsystems, Germany) and photographed at 100x magnification. The appropriate excitation and emission wave lengths to avoid non-specific signals were determined using EUB338 Cy3 and FITC labelled reference bacteria as 470–495 and 510–550 nm for FITC and 535–555 and 570–625 nm for Cy3. Images were analysed using the Leica LAS AF and Leica LAS AF lite (Leica Microsystems CMS GmbH, Wetzlar, Germany) imaging software packages.

## Supporting information

S1 Fig A DGGE gel (6% acrylamide) of PCR amplified products of DNA extracted from sodium hypochlorite washed male (HAM) and a female (HAF) *H*. *contortus* from each of three sheep. Samples were amplified using the universal bacterial 16S rRNA primers 338f (GC-clamp) and 518r. The gel was a portion of a 30–45% denaturing gradient.
(DOCX)

S2 Fig A DGGE gel (6% acrylamide) of PCR amplified products generated from the DNA extracted from manually collected worms from the abomasal mucosa and sodium hypochlorite washed worms from three sheep. The PCR was carried out using the universal bacterial 16S rRNA primers 338f (40bp GC clamp) and 518r. The gel was a portion of a 30–45% denaturing gradient.
(DOCX)

S3 Fig DGGE gels (6% acrylamide) of PCR amplified products of DNA extracted from samples collected during *H*. *contortus* larval culture from faeces. The DNA was amplified using the universal bacterial 16S rRNA primers 338f (40bp GC clamp) and 518r. DGGE gel 30–55% denaturing gradient: Lanes: A-fresh faecal sample, B-11 day old faecal sample mixed with vermiculite, C-larvae in water, D-larvae collected on a sieve, E-larvae collected at the bottom of a tube connected to the funnel, F-sheathed larvae washed with absolute ethanol, G-RO water, M-1 kb plus DNA ladder.
(DOCX)

S4 Fig Phylogenetic tree (ML method) of phylum Firmicutes ~1400bp 16S rRNA genes sequences from *H*. *contortus* using the primer set 27f and 1492r and reference gene sequences. Sequences belonging to order Clostridiales were compressed and represented as a triangle in the dendrogram. GenBank accession numbers of reference sequences are given before the reference cultures; (T) designates a type strain. Bootstrap values are shown at each node (percent of 500 replicates). HA: adult worms; HL: L3; HEF: eggs collected from faeces; HEM: eggs laid *in vitro*. The scale bar indicates 0.02 nucleotide substitutions per nucleotide position.
(DOCX)

S5 Fig Phylogenetic tree (ML method) of phylum Firmicutes ~1400bp bacterial 16S rRNA genes sequences from *H*. *contortus* using the primer set 27f and 1492r and reference gene sequences. Sequences belonging to families Leuconostocaceae, Streptococcaceae and Staphylococcaceae were compressed and represented as triangles in this dendrogram. GenBank accession numbers of reference sequences are given before the reference cultures; (T) designates a type strain. Bootstrap values are shown at each node (percent of 500 replicates). HA: adult worms; HL: L3; HEF: eggs collected from faeces; HEM: eggs laid *in vitro*. The scale bar indicates 0.02 nucleotide substitutions per nucleotide position.
(DOCX)

S6 Fig Phylogenetic tree (ML method) of phylum Firmicutes ~1000bp bacterial 16S rRNA genes sequences from *H*. *contortus* using the primer set 27f and 1040firmR and reference 16S rRNA gene sequences. GenBank accession numbers of reference sequences are given before the reference cultures; (T) designates a type strain. Bootstrap values are shown at each node (percent of 500 replicates). A: adult worms; L: L3; FE: eggs collected from faeces; ME: eggs laid *in vitro*. The scale bar indicates 0.02 nucleotide substitutions per nucleotide position.
(DOCX)

S1 Table Specificity, target and non-target species used to optimise bacterial probes used for fluorescence *in situ* hybridisation (FISH). (DOCX)

S2 Table Formamide concentrations for optimal hybridisation stringency of the bacterial species-, group- and class-specific fluorochrome-labelled probes used to identify bacteria in *H*. *contortus* by FISH. (DOCX)
