# Correction: The bacterial community associated with the sheep gastrointestinal nematode parasite *Haemonchus contortus*

**DOI:** 10.1371/journal.pone.0194378

**Published:** 2018-03-12

**Authors:** Gajenthiran Sinnathamby, Gemma Henderson, Saleh Umair, Peter Janssen, Ross Bland, Heather Simpson

The first author's name is spelled incorrectly. The correct name is: Gajenthiran Sinnathamby.
