# Supplementary material for: The bacterial community associated with the sheep gastrointestinal nematode parasite Haemonchus contortus
Source: PLoS One. 2018 Feb 8;13(2):e0192164. doi: 10.1371/journal.pone.0192164 (PMC5805237; doi:10.1371/journal.pone.0192164)
Supplement: S1 Fig — Samples were amplified using the universal bacterial 16S rRNA primers 338f (GC-clamp) and 518r. The gel was a portion of a 30–45% denaturing gradient. (DOCX) [file pone.0192164.s001.docx]

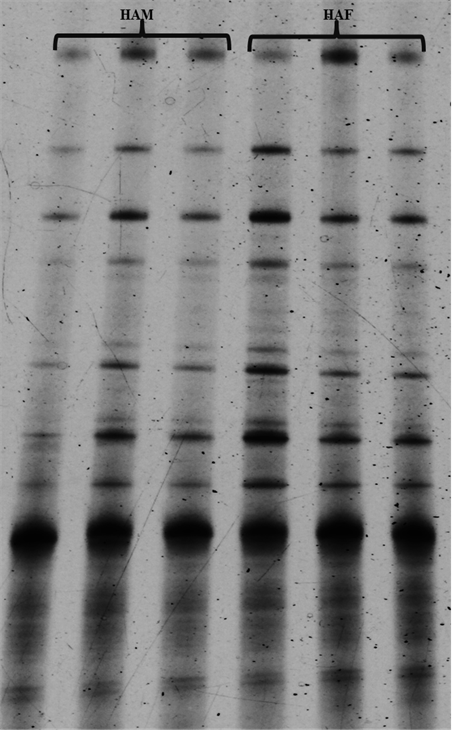


**Figure S1.** A DGGE gel (6% acrylamide) of PCR amplified products of DNA extracted from sodium hypochlorite washed male (HAM) and a female (HAF) *H. contortus* from each of three sheep. Samples were amplified using the universal bacterial 16S rRNA primers 338f (GC-clamp) and 518r. The gel was a portion of a 30-45% denaturing gradient.
