# Supplementary material for: The bacterial community associated with the sheep gastrointestinal nematode parasite Haemonchus contortus
Source: PLoS One. 2018 Feb 8;13(2):e0192164. doi: 10.1371/journal.pone.0192164 (PMC5805237; doi:10.1371/journal.pone.0192164)
Supplement: S2 Fig — The PCR was carried out using the universal bacterial 16S rRNA primers 338f (40bp GC clamp) and 518r. The gel was a portion of a 30–45% denaturing gradient. (DOCX) [file pone.0192164.s002.docx]

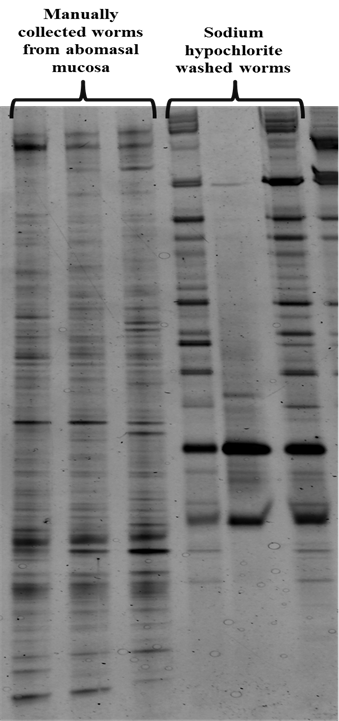


**Figure S2**. A DGGE gel (6% acrylamide) of PCR amplified products generated from the DNA extracted from manually collected worms from the abomasal mucosa and sodium hypochlorite washed worms from three sheep. The PCR was carried out using the universal bacterial 16S rRNA primers 338f (40bp GC clamp) and 518r. The gel was a portion of a 30-45% denaturing gradient.
