# Supplementary material for: The bacterial community associated with the sheep gastrointestinal nematode parasite Haemonchus contortus
Source: PLoS One. 2018 Feb 8;13(2):e0192164. doi: 10.1371/journal.pone.0192164 (PMC5805237; doi:10.1371/journal.pone.0192164)
Supplement: S3 Fig — The DNA was amplified using the universal bacterial 16S rRNA primers 338f (40bp GC clamp) and 518r. DGGE gel 30–55% denaturing gradient: Lanes: A-fresh faecal sample, B-11 day old faecal sample mixed with vermiculite, C-larvae in water, D-larvae collected on a sieve, E-larvae collected at the bottom of a tube connected to the funnel, F-sheathed larvae washed with absolute ethanol, G-RO water, M-1 kb plus DNA ladder. (DOCX) [file pone.0192164.s003.docx]

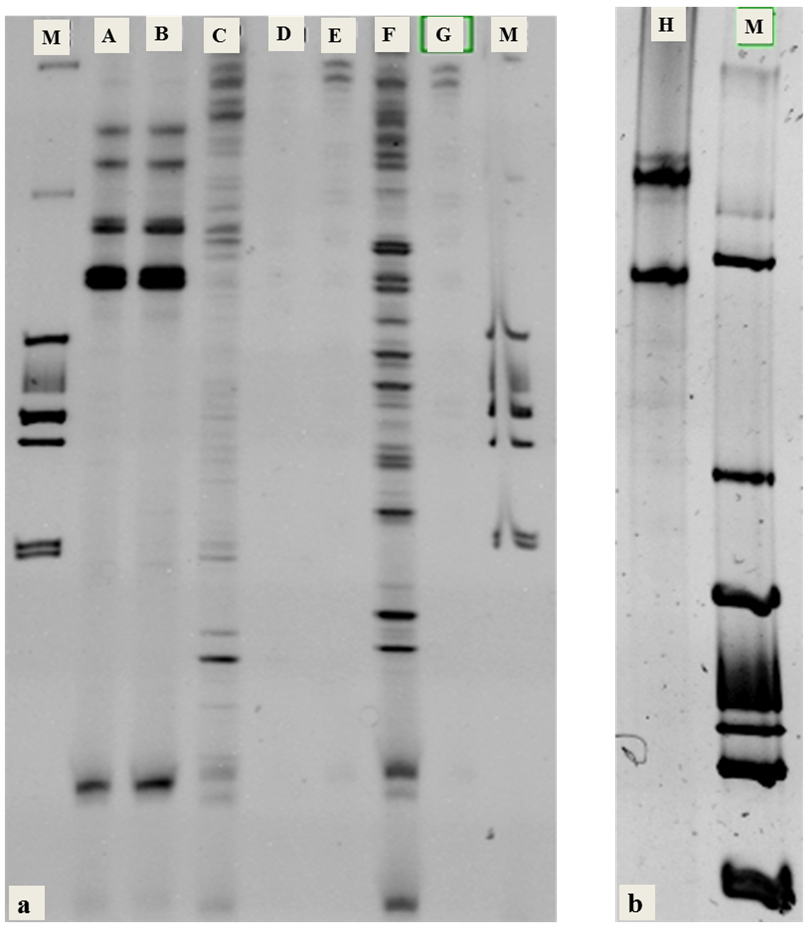


**Figure S3.** DGGE gels (6% acrylamide) of PCR amplified products of DNA extracted from samples collected during *H. contortus* larval culture from faeces. The DNA was amplified using the universal bacterial 16S rRNA primers 338f (40bp GC clamp) and 518r. DGGE gel 30-55% denaturing gradient**:** Lanes: A-fresh faecal sample, B-11 day old faecal sample mixed with vermiculite, C-larvae in water, D-larvae collected on a sieve, E-larvae collected at the bottom of a tube connected to the funnel, F-sheathed larvae washed with absolute ethanol, G-RO water, M-1 kb plus DNA ladder.
