# Supplementary material for: The bacterial community associated with the sheep gastrointestinal nematode parasite Haemonchus contortus
Source: PLoS One. 2018 Feb 8;13(2):e0192164. doi: 10.1371/journal.pone.0192164 (PMC5805237; doi:10.1371/journal.pone.0192164)
Supplement: S4 Fig — Sequences belonging to order Clostridiales were compressed and represented as a triangle in the dendrogram. GenBank accession numbers of reference sequences are given before the reference cultures; (T) designates a type strain. Bootstrap values are shown at each node (percent of 500 replicates). HA: adult worms; HL: L3; HEF: eggs collected from faeces; HEM: eggs laid in vitro. The scale bar indicates 0.02 nucleotide substitutions per nucleotide position. (DOCX) [file pone.0192164.s004.docx]

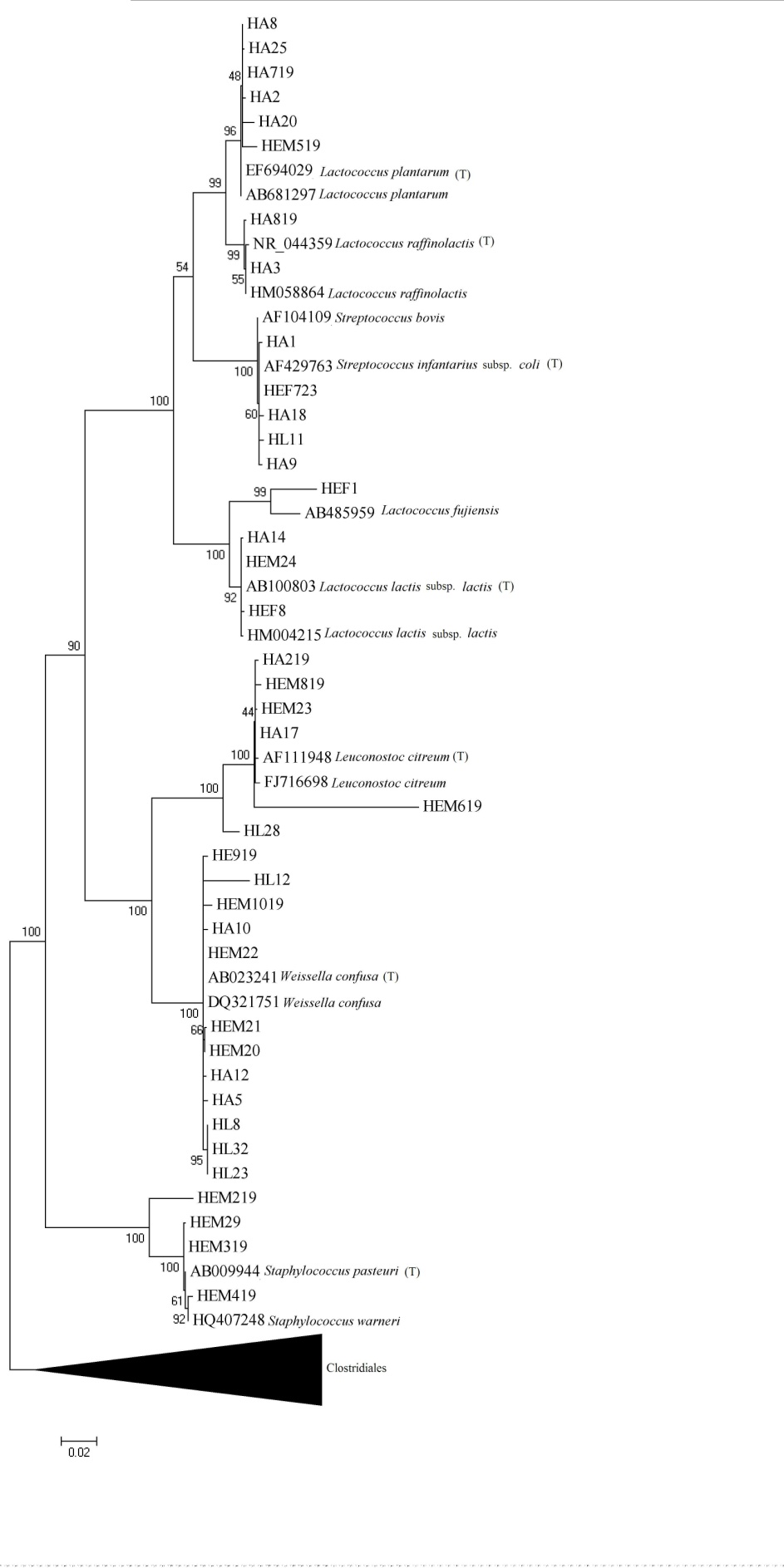


**Figure S4.** Phylogenetic tree (ML method) of phylum Firmicutes ~1400bp 16S rRNA genes sequences from *H. contortus* using the primer set 27f and 1492r and reference gene sequences. Sequences belonging to order Clostridiales were compressed and represented as a triangle in the dendrogram. GenBank accession numbers of reference sequences are given before the reference cultures; (T) designates a type strain. Bootstrap values are shown at each node (percent of 500 replicates). HA: adult worms; HL: L3; HEF: eggs collected from faeces; HEM: eggs laid *in vitro*. The scale bar indicates 0.02 nucleotide substitutions per nucleotide position.
