# Supplementary material for: The bacterial community associated with the sheep gastrointestinal nematode parasite Haemonchus contortus
Source: PLoS One. 2018 Feb 8;13(2):e0192164. doi: 10.1371/journal.pone.0192164 (PMC5805237; doi:10.1371/journal.pone.0192164)
Supplement: S5 Fig — Sequences belonging to families Leuconostocaceae, Streptococcaceae and Staphylococcaceae were compressed and represented as triangles in this dendrogram. GenBank accession numbers of reference sequences are given before the reference cultures; (T) designates a type strain. Bootstrap values are shown at each node (percent of 500 replicates). HA: adult worms; HL: L3; HEF: eggs collected from faeces; HEM: eggs laid in vitro. The scale bar indicates 0.02 nucleotide substitutions per nucleotide position. (DOCX) [file pone.0192164.s005.docx]

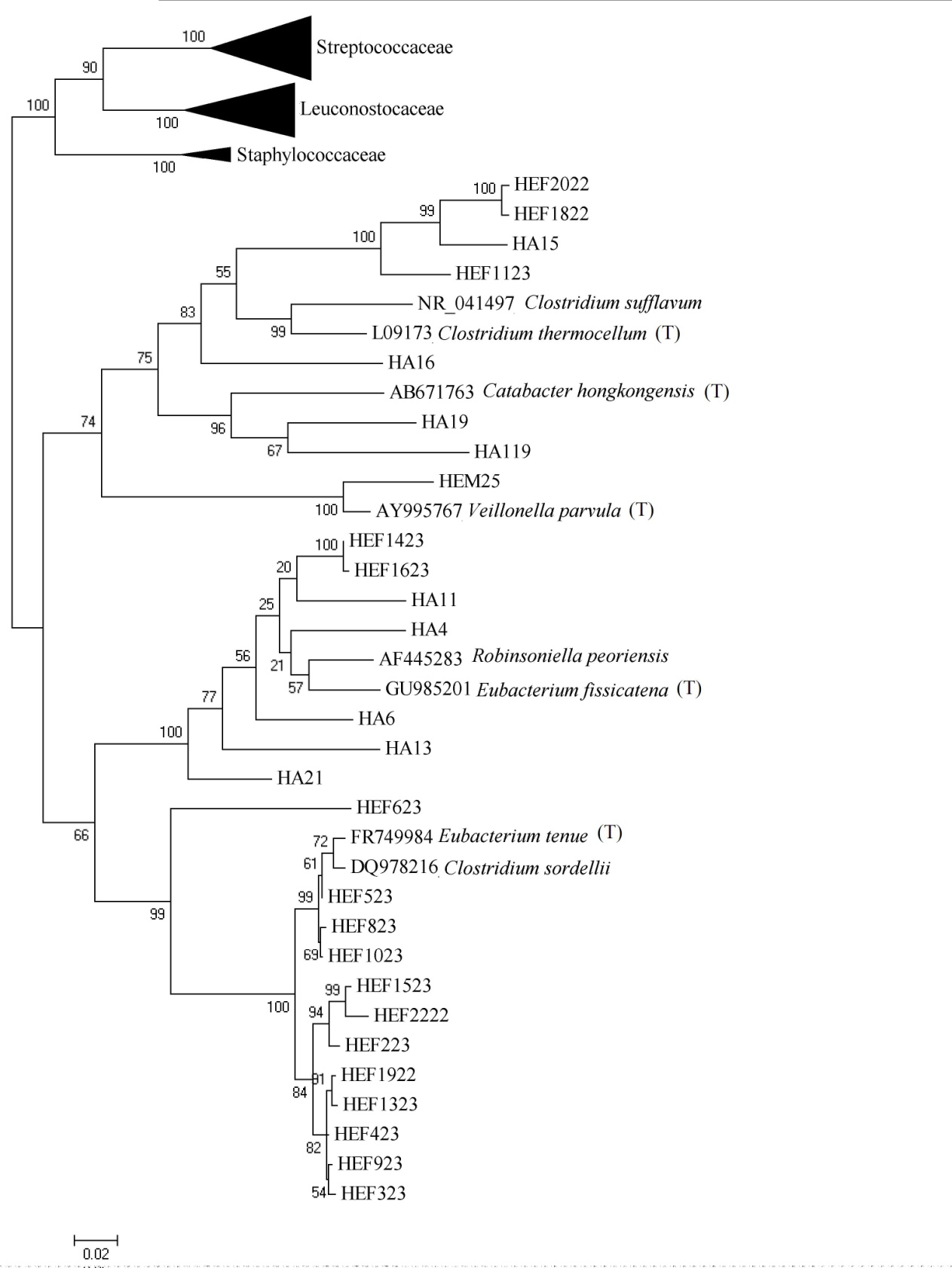


**Figure S5.** Phylogenetic tree (ML method) of phylum Firmicutes ~1400bp bacterial 16S rRNA genes sequences from *H. contortus* using the primer set 27f and 1492r and reference gene sequences. Sequences belonging to families Leuconostocaceae, Streptococcaceae and Staphylococcaceae were compressed and represented as triangles in this dendrogram. GenBank accession numbers of reference sequences are given before the reference cultures; (T) designates a type strain. Bootstrap values are shown at each node (percent of 500 replicates). HA: adult worms; HL: L3; HEF: eggs collected from faeces; HEM: eggs laid *in vitro*. The scale bar indicates 0.02 nucleotide substitutions per nucleotide position.
