# Supplementary material for: The bacterial community associated with the sheep gastrointestinal nematode parasite Haemonchus contortus
Source: PLoS One. 2018 Feb 8;13(2):e0192164. doi: 10.1371/journal.pone.0192164 (PMC5805237; doi:10.1371/journal.pone.0192164)
Supplement: S6 Fig — GenBank accession numbers of reference sequences are given before the reference cultures; (T) designates a type strain. Bootstrap values are shown at each node (percent of 500 replicates). A: adult worms; L: L3; FE: eggs collected from faeces; ME: eggs laid in vitro. The scale bar indicates 0.02 nucleotide substitutions per nucleotide position. (DOCX) [file pone.0192164.s006.docx]

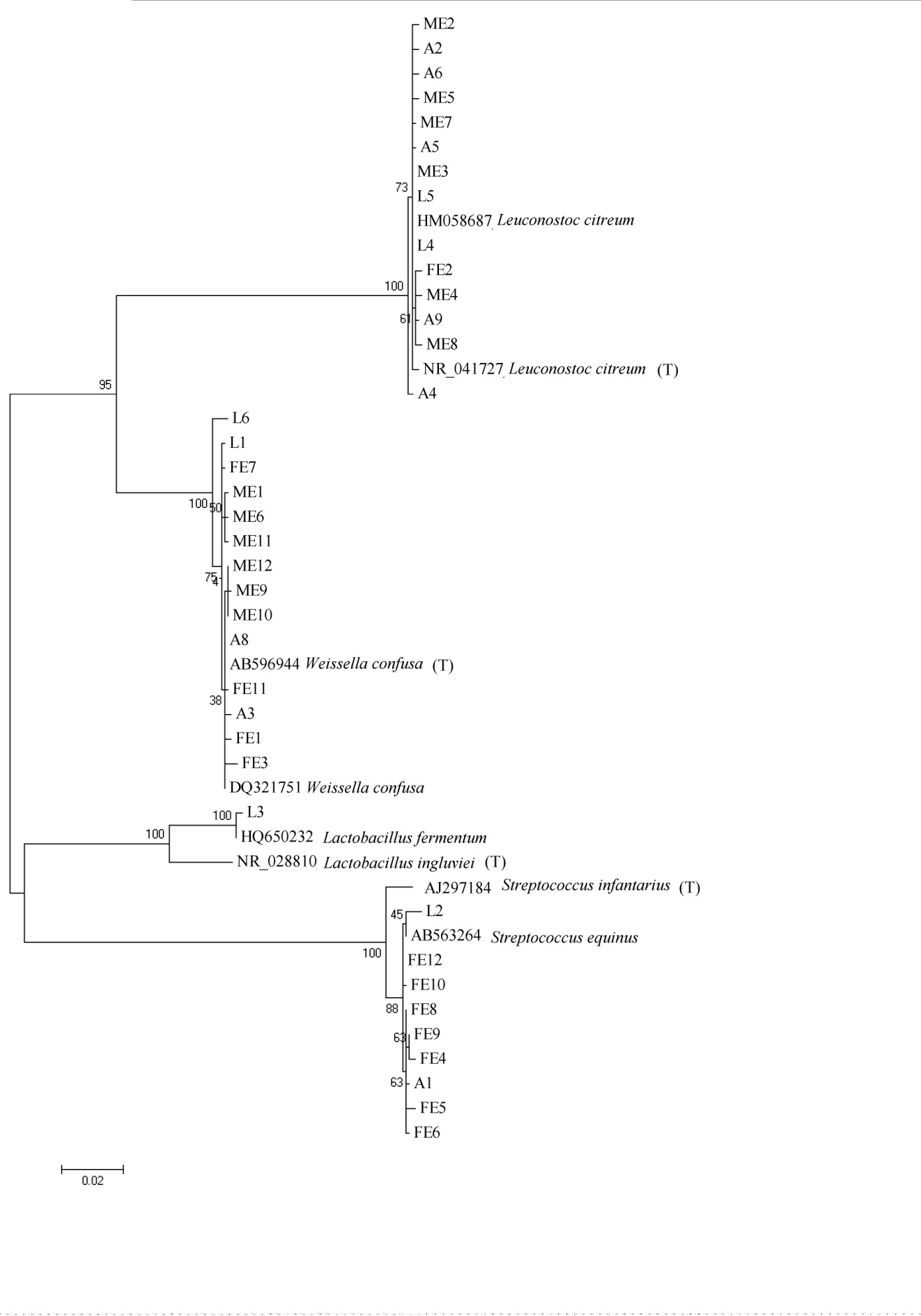


**Figure S6.** Phylogenetic tree (ML method) of phylum Firmicutes ~1000bp bacterial 16S rRNA genes sequences from *H. contortus* using the primer set 27f and 1040firmR and reference 16S rRNA gene sequences. GenBank accession numbers of reference sequences are given before the reference cultures; (T) designates a type strain. Bootstrap values are shown at each node (percent of 500 replicates). A: adult worms; L: L3; FE: eggs collected from faeces; ME: eggs laid *in vitro*. The scale bar indicates 0.02 nucleotide substitutions per nucleotide position.
