# Supplementary material for: The bacterial community associated with the sheep gastrointestinal nematode parasite Haemonchus contortus
Source: PLoS One. 2018 Feb 8;13(2):e0192164. doi: 10.1371/journal.pone.0192164 (PMC5805237; doi:10.1371/journal.pone.0192164)
Supplement: S1 Table — (DOCX) [file pone.0192164.s007.docx]

| Probe | Specificity | Target species | Non-target species |
| --- | --- | --- | --- |
| Lab158 | Most lactic acid bacteria | *Lactobacillus acidophilus*, *Enterococcus faecalis*, *Leuconostoc citreum*, *Weissella confusa* | *Weissella paramesenteroides, Streptococcus mutans, Lactococcus lactis* subsp. *lactis* |
| Wgp | *Weissella* sp. | *Weissella confusa* | *Streptococcus mutans, Enterococcus faecalis*, |
| S-G-WEI-0121-A-S-20 |  |  |  |
| Strc493 | Most *Streptococcus* sp., some *Lactococcus* sp. | *Streptococcus mutans,*  *Lactococcus lactis* subsp. *lactis* | *Lactobacillus acidophilus Enterococcus faecalis, Leuconostoc citreum, Weissella paramesenteroides Weissella confusa* |
| Alf73a | Alphaproteobacteria | *Aminobacter niigataensis* | *Streptococcus mutans*  *Weissella confusa* |
| Beta1 | Betaproteobacteria | *Comamonas testosteroni* |  |
| SteMa1_439 | Gammaproteobacteria | *Pseudomonas beteli* |  |

**Table S1.** Specificity, target and non-target species used to optimise bacterial probes used for fluorescence *in situ* hybridisation (FISH).
