# Supplementary material for: The bacterial community associated with the sheep gastrointestinal nematode parasite Haemonchus contortus
Source: PLoS One. 2018 Feb 8;13(2):e0192164. doi: 10.1371/journal.pone.0192164 (PMC5805237; doi:10.1371/journal.pone.0192164)
Supplement: S2 Table — (DOCX) [file pone.0192164.s008.docx]

**Table S2.** Formamide concentrations for optimal hybridisation stringency of the bacterial species-, group- and class-specific fluorochrome-labelled probes used to identify bacteria in *H. contortus* by FISH.

| Probe combination | Optimised formamide concentration (%) |
| --- | --- |
| EUB338 FITC and Lab158 Cy3 | 25 |
| EUB338 Cy3 and Lab158 FITC | 25 |
| EUB338 FITC and S-G-Wei-0121-a-S-20 Cy3 | 25 |
| EUB338 FITC and Wgp Cy3 | 25 |
| EUB338 FITC and Strc493 Cy3 | 35 |
| EUB338 Cy3 and Strc493 FITC | 35 |
| EUB338 FITC and ALF73a Cy3 | 5 |
| EUB338 FITC and Beta1 Cy3 | 10 |
| EUB338 FITC and SteMal_439 Cy3 | 10 |
